# Chemometrics-Assisted Raman Spectroscopy Characterization of Tunable Polymer-Peptide Hybrids for Dental Tissue Repair

**DOI:** 10.3389/fmats.2021.681415

**Published:** 2021-05-10

**Authors:** Paulette Spencer, Qiang Ye, Nilan J. B. Kamathewatta, Sarah K. Woolfolk, Brenda S. Bohaty, Anil Misra, Candan Tamerler

**Affiliations:** 1 Institute for Bioengineering Research, University of Kansas, Lawrence, KS, United States; 2 Department of Mechanical Engineering, University of Kansas, Lawrence, KS, United States; 3 Bioengineering Program, University of Kansas, Lawrence, KS, United States; 4 Department of Pediatric Dentistry, School of Dentistry, University of Missouri-Kansas City, Kansas City, MO, United States; 5 Department of Civil Engineering, University of Kansas, Lawrence, KS, United States

**Keywords:** raman chemical imaging, chemometrics, divisive clustering analysis, peptide-mediated remineralization, peptide-tethered adhesive, collagen, hybrid

## Abstract

The interfaces that biological tissues form with biomaterials are invariably defective and frequently the location where failure initiates. Characterizing the phenomena that lead to failure is confounded by several factors including heterogeneous material/tissue interfaces. To seamlessly analyze across these diverse structures presents a wealth of analytical challenges. This study aims to develop a molecular-level understanding of a peptide-functionalized adhesive/collagen hybrid biomaterial using Raman spectroscopy combined with chemometrics approach. An engineered hydroxyapatite-binding peptide (HABP) was copolymerized in dentin adhesive and dentin was demineralized to provide collagen matrices that were partially infiltrated with the peptide-functionalized adhesive. Partial infiltration led to pockets of exposed collagen–a condition that simulates defects in adhesive/dentin interfaces. The spectroscopic results indicate that co-polymerizable HABP tethered to the adhesive promoted remineralization of the defects. The spatial distribution of collagen, adhesive, and mineral as well as crystallinity of the mineral across this heterogeneous material/tissue interface was determined using micro-Raman spectroscopy combined with chemometrics approach. The success of this combined approach in the characterization of material/tissue interfaces stems from its ability to extract quality parameters that are related to the essential and relevant portions of the spectral data, after filtering out noise and non-relevant information. This ability is critical when it is not possible to separate components for analysis such as investigations focused on, *in situ* chemical characterization of interfaces. Extracting essential information from complex bio/material interfaces using data driven approaches will improve our understanding of heterogeneous material/tissue interfaces. This understanding will allow us to identify key parameters within the interfacial micro-environment that should be harnessed to develop durable biomaterials.

## INTRODUCTION

### Clinical Need

Untreated dental caries of permanent teeth impact 2.3 billion people across the globe and more than 530 million children suffer from untreated dental caries of primary teeth (WHO, 2020). Tooth decay is the most common chronic childhood disease (Garvin, 2021).

The most popular material for the repair of lost or damaged tooth structure is dental composite (Ferracane, 2017; Eltahlah et al., 2018), but composite fails at a rate 2–3.5 times the rate of dental amalgam (Ferracane, 2013; Schwendicke et al., 2016; Afrashtehfar et al., 2017; Makvandi et al., 2018). The cycle of repeated composite-restoration replacements is a pernicious problem–each replacement risks pulpal injury, increased tooth weakness, and eventually, tooth loss (Opdam et al., 2016). Patients at high risk for caries, such as the 4 million United States children (Palmer, 2013) and more than 100 million adults (National Center for Health Statistics (US), 2007) who do not receive regular dental care, are particularly vulnerable to composite-restoration failure and the downward spiral associated with frequent replacements (Kopperud et al., 2015; Schwendicke et al., 2016).

### Composite Restoration Failure

**FIGURE 1 |:**
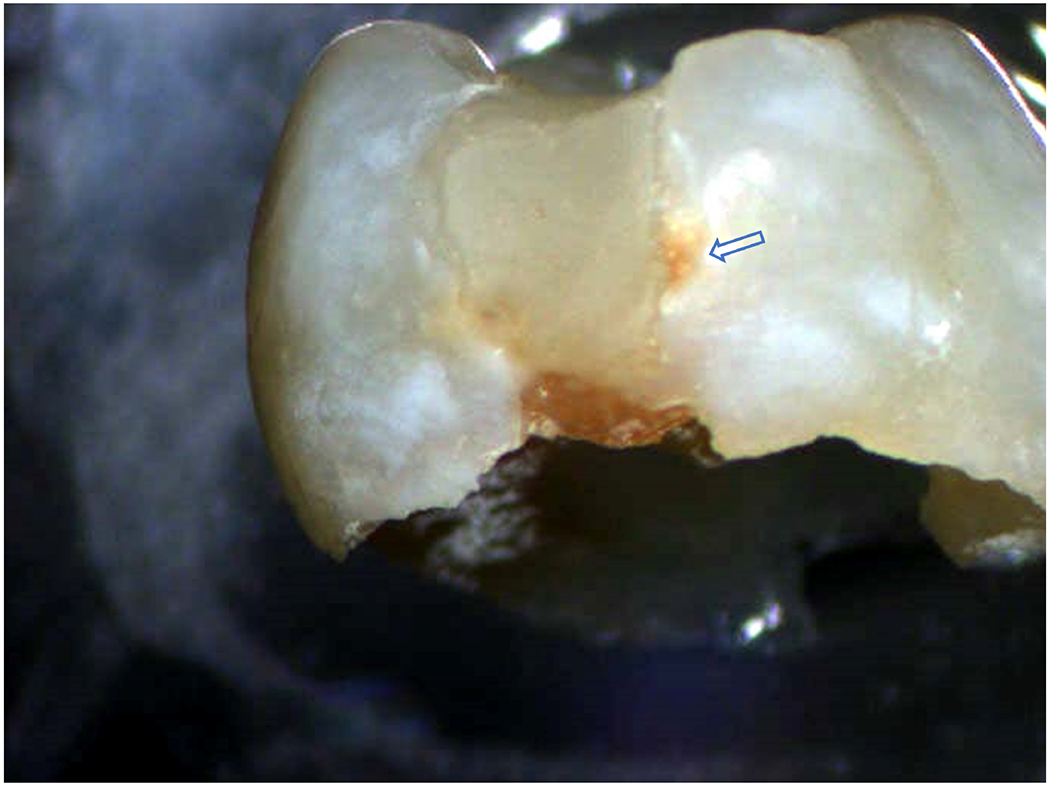
Exfoliated primary molar with Class II composite restoration. Brown stain along the interface between the composite material and tooth structure indicates recurrent decay at the material/tooth interface.

The leading cause of composite-restoration failure is recurrent marginal decay (Figure 1; Stewart and Finer, 2019). Unlike amalgam, composite lacks the inherent capability to seal gaps at the interface between the restorative material and tooth structure. The low-viscosity adhesive that bonds the composite to the tooth is intended to seal this interface, but the adhesive seal to dentin is fragile—it is readily damaged by acids, enzymes, and oral fluids. The fragility of the adhesive/dentin seal is traced to the hybrid layer.

**FIGURE 2 |:**
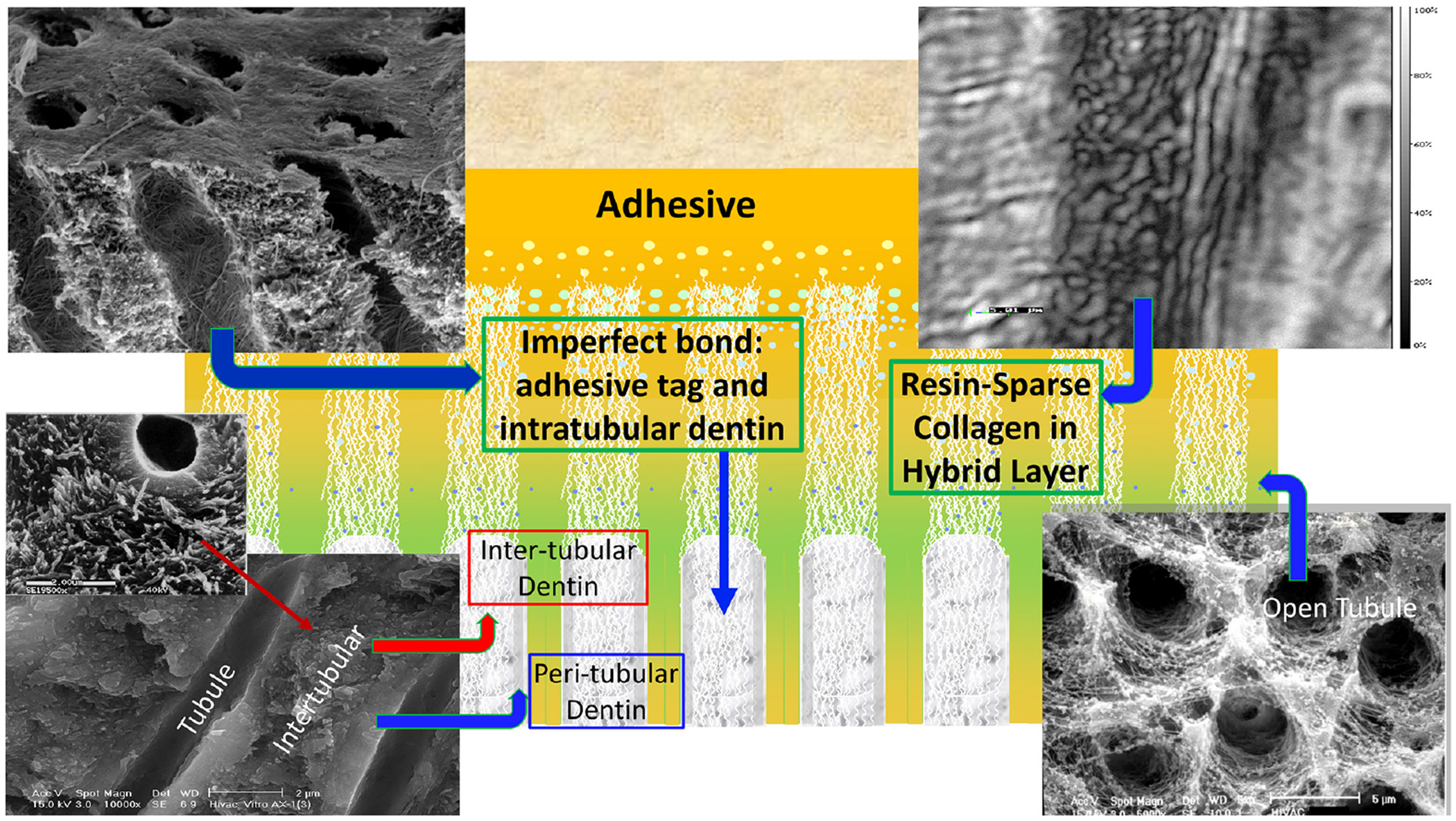
A schematic depicting cross-section of adhesive infiltrating dentin. Areas of resin-sparse collagen within the hybrid layer are illustrated as well as imperfect adhesive bonds to intra-, inter-, and peritubular dentin.

The ideal hybrid layer is described as a 3D polymer/collagen construct that provides a continuous and stable link between the bulk adhesive and mineralized dentin, but this ideal is not achieved—the hybrid layer retains pockets of resin-sparse collagen that are at inherent risk of degradation (Spencer and Swafford, 1999; Spencer and Wang, 2002; Wang and Spencer, 2005; Spencer et al., 2006; Kermanshahi et al., 2010; Liu et al., 2011; Pashley et al., 2011; Tjaderhane et al., 2013). The pockets of resin-sparse collagen are readily infiltrated by bacterial enzymes and acids—hydrolysis provoked by these agents leads to a breach of the interfacial seal. Once the seal is breached, acids and enzymes permeate defects in the adhesive bond to the inter-, intra-, and peritubular dentin (Figure 2). Bacteria and bacterial by-products traverse these imperfections, destroying tooth structure and eroding adhesive in its path.

### Bioactive Strategies to Increase Stability of the Hybrid Layer

A variety of strategies have been explored to increase the stability and durability of the hybrid layer. One particularly promising strategy is remineralization—voids are filled with mineral to reduce the permeability of the hybrid layer.

Several groups have proposed biomimetic remineralization as a novel approach to increase the durability of the hybrid layer (Lin et al., 2016; Nurrohman et al., 2016; Panseri et al., 2016; Moussa and Aparicio, 2019; Ye et al., 2021). As an example, a calcium phosphate polymer-induced liquid precursor (Ca/P-PILP) was used to promote the repair of artificially induced, carious dentin (Bacino et al., 2019; Chen et al., 2020). The PILP promoted the recovery of both the structure and mechanical properties of the damaged dentin (Saeki et al., 2017; Saxena et al., 2019).

Our group used peptides to promote the remineralization of defective dentin matrices (Ye et al., 2017). With this peptide-mediated approach, we demonstrated that calcium phosphate minerals were formed at the deficient adhesive/dentin interface. While these results were promising, self- assembled peptide binding at this interface could lead to peptide diffusion and limited active conformations due to the challenges associated at the hybrid layer. To address this limitation, we prepared a peptide-functionalized adhesive to provide *in situ* presentation of the bioactive cues at the adhesive/dentin interface. The hydroxyapatite binding peptides (HABP) were synthesized using oligomeric spacers to tether them to a methacrylic acid (MA) as co-polymerizable peptide in the dental adhesive. Demineralized dentin collagen was partially infiltrated with the peptide-functionalized adhesive—the adhesive/collagen hybrids mimic adhesive/dentin interfaces that contain defects such as pockets of resin-sparse collagen (Spencer and Swafford, 1999; Spencer et al., 2000; Wang et al., 2007).

### *In situ* Characterization

The adhesive/collagen hybrids contain vastly different structures and heterogeneous composition. To seamlessly analyze across these complex, heterogeneous interfaces presents numerous analytical challenges. Regardless of the challenges, *in situ* characterization is central to identifying factors key to designing a peptide-functionalized adhesive that promotes remineralization of defective dentin matrices.

Raman microspectroscopy has steadily evolved to become a versatile method for *in situ* structural characterization of the adhesive/dentin interface (Suzuki et al., 1991; Van Meerbeek et al., 1993; Lemor, 1997; Wang and Spencer, 2002b; Spencer and Wang, 2007; Wang et al., 2007). The Raman spectrum probes the chemical structure and provides a distinct “fingerprint” of the molecules present in a sample and can be used for both qualitative identification and quantitative determination. This vibrational spectroscopy technique has many advantages—it is non-destructive, non-invasive, and multi-dimensional results are obtained in minutes. This technique provides suitable sensitivity for analyzing mineral polymorphism and crystallinity (Wang et al., 1994, 2006; Ohsaki et al., 1995; Tsuda et al., 1996; Kontoyannis et al., 1997).

Usually, hyperspectral Raman imaging consists of thousands of spectra gathered in a data-cube, i.e., a three-dimensional matrix with two spatial dimensions (x, y) and one spectral dimension (λ). It can be challenging to extract useful information from these large, complex datasets (Paudel et al., 2015; Rebiere et al., 2018). If an individual component can be uniquely identified by a spectral band, analysis of its band intensity is relatively straightforward and such analysis is used to identify its location in the sample. This univariate analysis is considered the simplest and most frequently used method and, in many cases, can provide sufficient information and reliable predictability (Wieliczka et al., 1997; Salzer et al., 2000; Wang and Spencer, 2002a,b; Wang et al., 2006; Sinjab et al., 2017).

Univariate analysis is generally not sufficient when spectral bands partially overlap due to interference from other components in the specimen. Under these circumstances, the analysis of spectra datasets requires additional procedures to extract useful information (Calvo et al., 2018). Multi-variate analysis, i.e., chemometrics can be used to address these challenges and to identify important details hidden in the structure. Common multivariate methods include principal component analysis, partial least square regression (PLS), classical least square (CLS), multivariate curve resolution (MCR), partial least square discriminant analysis (PLS-DA), and so forth (Paudel et al., 2015; Dina et al., 2018; Pisapia et al., 2018; Rebiere et al., 2018; Fang et al., 2020).

In this study, divisive cluster analysis was used in combination with micro-Raman spectroscopy to analyze details hidden in the structure of the peptide-functionalized adhesive/collagen hybrid before and after peptide-mediated remineralization. The spatial distribution and relative concentration of the components as well as crystallinity of the mineral produced as a result of peptide-mediated remineralization were determined. To analyze the earliest stage of peptide-mediate mineralization at the collagen interface, type I collagen model and co-polymerizable hydroxyapatite-binding peptide were studied using atomic force microscopy as a complementary technique to micro-Raman spectroscopy. Our results demonstrate that the structural details revealed by Raman spectroscopy combined with chemometrics enhance our understanding of the composition and mineralization capability at the challenging biohybrid interfaces.

## MATERIALS AND METHODS

### Materials

The following components were obtained from Sigma-Aldrich (St. Louis, MO): 2-hydroxyethyl methacrylate (HEMA), triethylene glycol dimethacrylate (TEGDMA), methacrylic acid (MA), camphoroquinone (CQ), ethyl-4-(dimethylamino) benzoate (EDMAB), diphenyliodonium hexafluorophosphate (DPIHP), mono(2-methacryloyloxy)ethyl succinate (MMES), dodecyltrichlorosilane, chlorhexidine digluconate (CHX), N,N-Dimethylformamide (DMF), dichloromethane (DCM), and N-methyl morpholine (NMM). γ-methacryloxypropyl trimethoxysilane (MPS) was used as received from MP Biomedicals (Solon, OH). Rink amide resin, Fmoc-amino acid building blocks and 2-(1H-benzotriazole-1-yl)-1,1,3,3-tetramethyluranium hexafluorophosphate (HBTU) were purchased from AAPPTec LLC (Louisville, KY). All chemicals were used as received without further purification.

### Co-polymerizable Hydroxyapatite-Binding Peptide

We demonstrated that hydroxyapatite-binding peptide (HABP) having CMLPHHGAC sequence can selectively self-assemble on hydroxyapatite minerals, control mineralization nucleation and growth kinetics, and guide the nano- to micro-structure organization of the mineral in the absence of cells (Gungormus et al., 2008; Ye et al., 2017). We developed spacer design to conjugate peptides to the methacrylate-based monomers by providing reactive groups for monomer conjugation as well as sufficient length and flexibility to preserve the peptide’s properties once the polymer has cured (Xie et al., 2020). The co-polymerizable hydroxyapatite-binding peptide, MMES-KGGG_HABP or MA-KGGG-HABP was synthesized in our lab (with KGGG as the spacer), via an amidation reaction between the free amine group (peptide) and carboxylic acid group of the monomers (Xie et al., 2020). Briefly, Fmoc-resin-bound peptide with a spacer was first synthesized through Fmoc-chemistry using a solid-phase peptide synthesizer (AAPPTEC Focus XC). Upon peptide-chain completion (including spacer sequence), MA or MMES, NMM, and HBTU were added to react with the Fmoc-resin-bound peptide in DMF at 23 ± 2°C overnight under constant gentle rotation. After the conjugation reaction, the Fmoc-resin-bound product was washed sequentially with DMF, DCM, acetone, and ethanol. The crude co-polymerizable HABP was then cleaved from the Fmoc resin and purified on a HPLC system (Waters Corp., Milford, MA, United States) equipped with a Luna^®^ column packed with 10 μm C18 silica (250 × 4.6 mm, Phenomenex Inc., Torrance, CA, United States). The co-polymerizable peptide was lyophilized, and stored at −20°C. Crude peptides were also cleaved from the resin and then purified. The purified peptide fractions were combined and lyophilized (Xie et al., 2020).

### Preparation of Peptide-Functionalized Adhesive Formulation

The model hydrophilic adhesive consisted of 2-hydroxyethylmethacrylate (HEMA), triethylene glycol dimethacrylate (TEGDMA) and (trimethoxysilyl) propyl methacrylate (MPS) with a mass ratio of 8/1/1 (HEMA/TEGDMA/MPS). This model hydrophilic adhesive system has been developed and optimized for peptide engineering in our previous investigations (Ye et al., 2011; Abedin et al., 2014; Xie et al., 2020). The following photoinitiators (all from Aldrich, Milwaukee, WI, United States) were used: camphoroquinone (CQ), ethyl-4-(dimethylamino) benzoate (EDMAB) and diphenyliodonium hexafluorophosphate (DPIHP). The amounts of photosensitizer, coinitiator amine and iodonium salt were fixed at 0.5 mass% with respect to the total amount of monomer (Guo et al., 2008; Ye et al., 2009; Song et al., 2014). The resin mixtures were prepared in a brown glass vial under amber light. Continuous shaking and sonication for 48 h were required to yield well-mixed homogenous resin solutions (Song et al., 2016). The hydrophilic adhesive formulation was mixed with 10 mass per-cent co-polymerizable hydroxyapatite-binding peptide, e.g., MMES- KGGG_HABP, and diluted with ethanol in a weight ratio of 80/20.

### Dentin Demineralization

**SCHEME 1 |:**
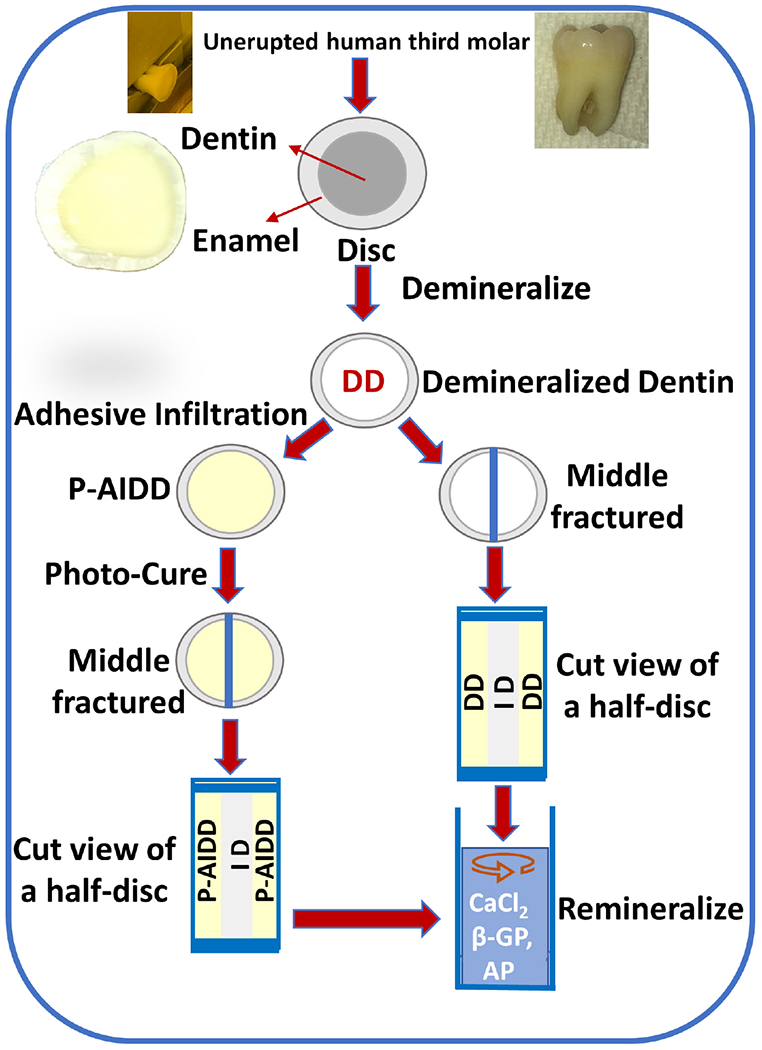
Flow chart of the steps involved in obtaining demineralized dentin (DD) samples and peptide-functionalized adhesive-infiltrated demineralized dentin (P-AIDD) samples and their remineralization protocol.

Extracted unerupted human third molars (*n* = 8) were collected and stored at 4°C in 0.9% w/v NaCl containing 0.002% sodium azide (these teeth would otherwise be discarded, no patient identifiers are associated with the teeth and thus, this is not considered human subjects research). The occlusal 1/3 of the crown was sectioned perpendicular to the long axis of a human molar using a water-cooled low-speed diamond saw (Scheme 1). The retrieved disc-shaped specimens are ~1-mm thick. The dentin surface was polished on 600-, 1, 200-, and 2000-grit silicon carbide papers, respectively, under running water, followed by sonication for 10 min between each grit to remove cutting debris. The dentin discs with surrounding enamel were etched for 6 h with 10% phosphoric acid. This etching protocol is different from the commonly employed clinical treatment, i.e., 20 s etching with 34% phosphoric acid. Our objective was to provide a substantial and uniform zone of completely demineralize dentin—the resultant collagen network was used to monitor the remineralization effect. To evaluate the extent of demineralization, randomly selected specimens were fractured in liquid nitrogen and the exposed fracture surface was characterized using micro-Raman spectroscopy. The depth of demineralization was usually 100–200 microns for the 1 mm thick dentin disc, and the etched zone was uniform and presented as a completely demineralized collagen network. There was no detectable mineral loss in the region identified as intact dentin (ID).

### Preparation of Test Group and Control Samples

#### Test Group Samples

Following demineralization, randomly selected dentin disc specimens were immersed in the liquid resin, i.e., the peptide-functionalized adhesive, and stored for 48 h in the dark. Dark storage for 48 h allowed time for full infiltration of the liquid resin throughout the demineralized dentin zone, that means the infiltration depth reached 100–200 microns for the 1-mm thick disc specimens. At 48 h, the sample was removed from the liquid resin and a stream of air was blown across the surface to remove excess resin. The sample was polymerized using a halogen light curing unit of irradiance 550 mW/cm^2^ for 60 s. The specimen was stored in the dark at room temperature for 24 h to provide time for post-cure polymerization. The test group samples are noted as (Peptide-functionalized-adhesive)-Adhesive-Infiltrated Demineralized Dentin (P-AIDD).

#### Control Specimens

Randomly selected demineralized dentin discs were used as the control group. The control specimens are noted as demineralized dentin (DD).

### Remineralization

The mineralization solution (2X) was prepared using 48 mM CaCl_2_ and 28.8 mM β-Glycerophosphate (β-GP) in 50 mM Tris-HCl buffer, pH 7.4 (Ye et al., 2017). The test group and control samples were fractured in the middle, and then incubated with mineralization solution for 72 h at 37°C with a daily change of fresh biomineralization solution. The remineralization reaction was initiated by adding alkaline phosphatase (AP, Thermo Scientific) at a final concentration of 1.4 × 10^−6^ g/mL. In this reaction, the enzyme alkaline phosphatase hydrolyses the organic phosphate compound to PO_4_^−3^. Shaking at 100 rpm continued throughout the exposure of the samples to the mineralization solution.

### Raman Spectroscopy

The demineralized dentin specimens (as control) and P-AIDD specimens (as test group samples) before and after remineralization were imaged using a LabRAM ARAMIS Raman microscope (HORIBA Jobin Yvon, Edison, NJ, United States). This Raman spectrometer was equipped with a HeNe laser (λ = 633 nm, a laser power of 17 mW) as an excitation source. The samples were mounted in a computer-controlled, high-precision x-y stage. Raman spectra were acquired under these instrument conditions: 200 μm confocal hole, 150 μm wide entrance slit, 600 g/mm grating, 15 s spectra acquisition time, four acquisitions per cycle, and 50X long working distance objective Olympus lens. With the assistance of HORIBA’s EasyNav™ package, Raman spectra were acquired over a range of 300–1,800 cm^−1^ and data processing was performed using LabSPEC 6 software (HORIBA Jobin Yvon, Edison, NJ, United States). In this work, two-dimensional micro-Raman mapping/imaging was used to determine the spatial relationships and distribution of the functional or chemical groups. The spectra were collected from the defined area at regular intervals of 15 μm in both X and Y planes. At least four rectangular areas from test group or control group samples were imaged and submitted to spectral analysis.

### Chemometrics Analysis

A number of multivariate chemometric methods powered by Eigenvector Research Inc. are fully integrated in LabSPEC 6’s Multivariate Analysis (MVA) module (HORIBA Jobin Yvon, Edison, NJ). Divisive Clustering Analysis (DCA) was selected for this study to classify and group related spectra. Using DCA the specimen was divided into chemically different regions with successively increasing detail. A rectangular area of the surface was imaged and submitted to DCA, which includes a statistical pattern to derive the independent clusters. Principal Components Analysis decomposed the data set into a bilinear model of linear independent variables, the so-called principal components (PCs). DCA initially assumes that all spectra belong to a class. Through an iterative process, spectra are divided into the specified number of groups, so that spectra belonging to the same group are similar to each other. The results are class memberships for each spectrum. Once analysis is completed the average spectrum of each class will be displayed together with the analysis statistics. The class membership images reveal which class each pixel spectrum has been assigned. For each test group or control group, at least four rectangular areas are imaged and submitted to DCA, and a representative image was presented.

### Further Spectral Analysis

The average spectra were calculated for each cluster, which can be used to provide information on peak parameters (e.g., position, width, amplitude, area) and component distribution in Raman images. At this point, the components were analyzed as follows.

#### Relative Mineral Concentration

Mineral-to-matrix ratio (MMR) was inferred from the ratio of the intensities of the peaks at 960 cm^−1^ (phosphate) and 1,460 cm^−1^ (CH_2_ wagging, amide II). The index was representative of the maximum relative degree of mineralization.

#### Crystallinity

Crystallinity was evaluated based on the full width at half maximum (FWHM) of the *v*1 phosphate band at 960 cm^−1^. This index expressed the crystallographic or relative atomic order, since narrower peaks suggest less structural variation in bond distances and angles. In general, the narrower the spectral peak width, the higher the degree of mineral crystallinity.

##### Gradient in mineral content (GMC), or carbonate content of mineral crystallites:

GMC was based on the ratio of the relative peak heights of 1,070 cm^−1^ (carbonate) to 960 cm^−1^ (phosphate). This ratio provided an assessment of carbonate substitution for phosphate.

#### Adhesive Infiltration (AdhI)

The ratio of the relative peak heights of 1,450/1,667 was used to assess the extent of adhesive infiltrated into the collagen matrix. The peak at 1,450 cm^−1^ is assigned to the CH_2_ group of methacrylate-based adhesive, and the peak at 1,667 cm^−1^ is assigned to amide I associated with collagen.

Statistical analysis was used to identify significant differences in the means. The results (FWHM, GMC, Relative mineral concentration, and adhesive infiltration) were analyzed using one-way analysis of variance (ANOVA) together with Tukey’s test at α = 0.05 (Microcal Origin Version 8.0, Microcal Software Inc., Northampton, MA, United States).

### Analyses of Earliest Stage Mineralization

To assess the earliest stages of peptide-mediated mineralization at the interface between collagen and co-polymerizable hydroxyapatite-binding peptide (MAHABP), type I collagen model specimens prepared by spin-coating were analyzed using atomic force microscopy. Aqueous collagen solution (type I) from rat tail (Sigma C3867) mixed with 4mg/ml HABP or MAHABP was coated on round glass coverslip (12 mm diameter, 26023, Ted Pella Inc.) with a WS-400E-6NPP-LITE spin coater (Laurell Technologies, North Wales, PA) at speed of 1000 RPM. The resultant thin film was incubated with mineralization solution for 20 or 40 min, and then analyzed using AFM and Raman. The AFM images were obtained with a Multimode 8 HR scanning probe microscope (Bruker Corporation, Camarillo, CA) operated in tapping mode under ambient conditions (24 ± 2°C, 40% ± 5% RH). Tapping mode etched silicon probes (Prod No.: RTESPA-300, Bruker) were used, having a resonant frequency of about 285 KHz. The length and thickness of the probes were 115–135 μm and 38–42 μm, respectively. Images of each sample were recorded with Nanoscope 8.15 software and analyzed with the Nanoscope Analysis 2.0 software. Related, but separate samples of type I collagen model specimens mixed with MAHABP were analyzed using Raman.

## RESULTS

**FIGURE 3 |:**
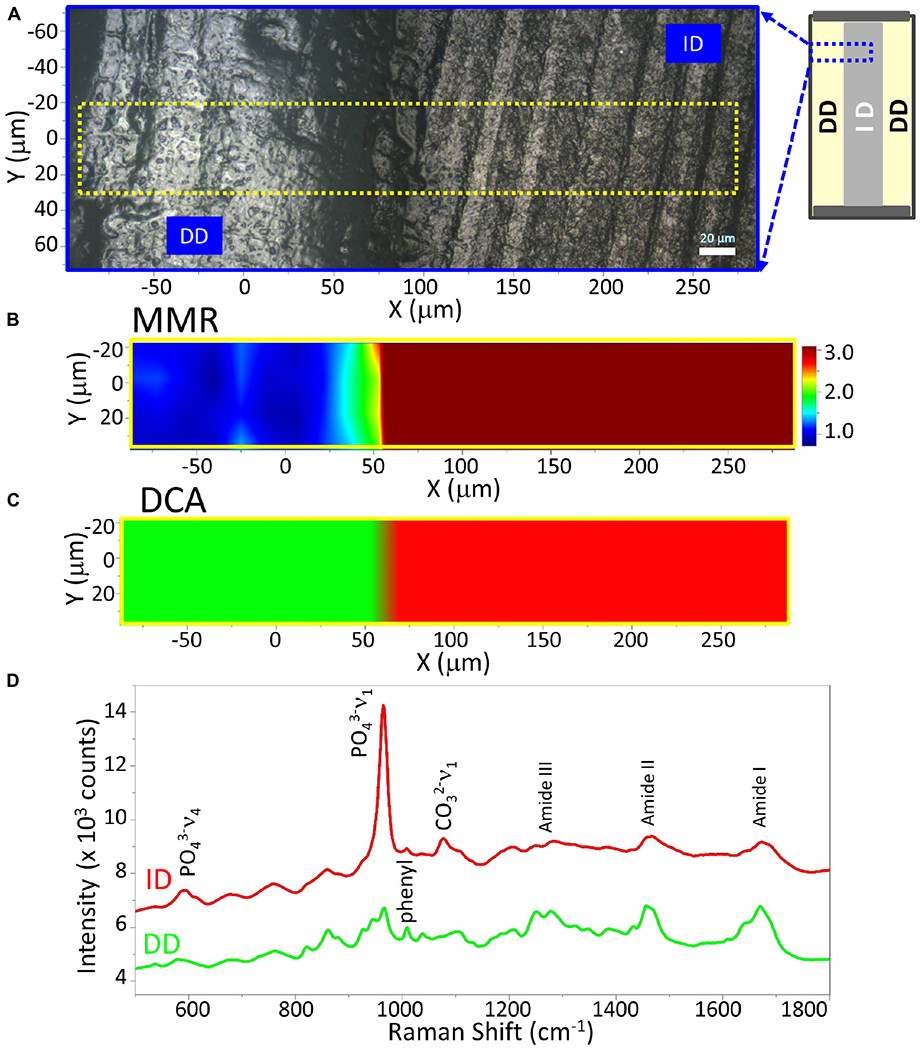
Raman analysis of a representative demineralized dentin disc (control). **(A)** Light micrograph marked with a X-Y scan area. Scan bar is 20 μm. **(B)** μ-Raman mineral map (MMR) of the X-Y scan area. Blue represents the lowest ratio, while red represents the highest. **(C)** K-means clustering map (DCA) of the Raman profile of the same scan area. Two distinguished classes are identified, e.g., intact dentin (ID) and demineralized dentin (DD) region. **(D)** μ-Raman spectra of two classes, corresponding to ID and DD regions.

Two-dimensional XY Raman imaging is acquired from the scan area of the demineralized dentin specimen shown in the light micrograph (Figure 3A). Spectra are acquired at points across the DD (left, Figure 3A) and ID (right, Figure 3A). The most straightforward method of spectral analysis is to create functional group maps based on band intensities, band areas, or band ratios (univariate analysis). The micro-Raman mineral map (Figure 3B) is a “false-color composite” image generated from band ratios 960 cm^−1^ (PO_4_^−3^)/1,460 cm^−1^ (CH_2_ wagging, amide II). In this image, intact dentin is dark red while demineralized dentin is blue.

A color representation of the K-means cluster analysis, corresponding to the same mapping zone shown in Figure 3B, is presented in Figure 3C. This technique was used to divide the scanned area into chemically different regions in successively increasing detail. The cluster analysis displayed two well-distinguished classes, and their multivariate maps were displayed in the overlay mode confirming the result from the univariate analysis, e.g., the demineralized dentin (in green) on the left and intact dentin (in red) on the right. The cluster centroid spectra from the two principal components are derived (Figure 3D), which denote the center of the mean of the clusters. For each point of analysis, all spectra described for each cluster were averaged to obtain the mean cluster spectrum. The Raman spectral features of ID associated with dentin mineral are phosphate (PO_4_^−3^) and carbonate (CO_3_^−2^) at 960 cm^−1^ and 1,070 cm^−1^, respectively. These mineral-derived bands diminished in the demineralized dentin spectra, where spectral features assigned to dentin collagen became obvious, such as 1,003 cm^−1^ (C-C in phenyl group), 1,667 cm^−1^ (*C* = O, amide I), 1,460 cm^−1^ (CH_2_ wagging, amide II), and doublet bands from 1,215 to 1,310 cm^−1^ (N-H, amide III) (Figure 3D).

**FIGURE 4 |:**
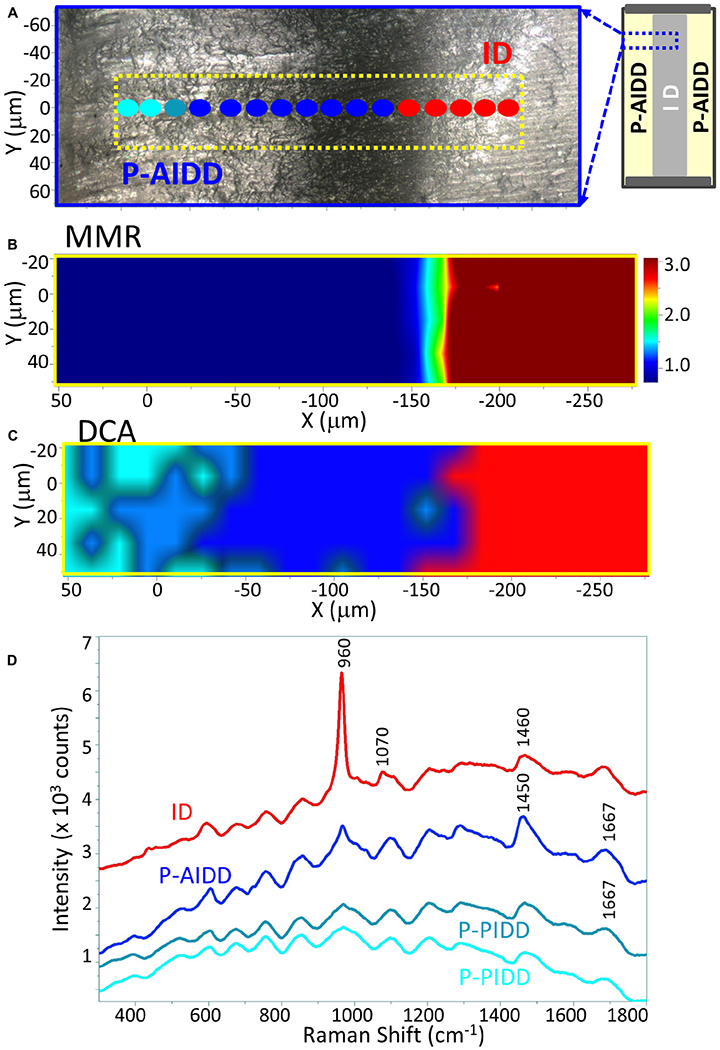
Raman analysis of a representative peptide-functionalized adhesive-infiltrated demineralized dentin (P-AIDD) specimen. **(A)** Light micrograph marked with a X-Y scan area, **(B)** μ-Raman mineral map (MMR) of the X-Y scan area. **(C)** K-means clustering map (DCA) of the Raman profile of the same scan area. Four classes are identified, including intact dentin (ID), peptide-functionalized adhesive-infiltrated demineralized dentin (P-AIDD) and two peptide-functionalized partially-infiltrated demineralized dentin (P-PIDD) regions with similar spectral features. **(D)** Raman spectra from the four classes.

The representative Raman images of peptide-functionalized adhesive-infiltrated demineralized dentin (P-AIDD) specimens are shown in Figure 4. For ease of comparison, Figure 4 is presented in a manner similar to Figure 3. Two-dimensional XY Raman imaging is acquired from the scan area shown in the light micrograph (Figure 4A). The micro-Raman mineral map (Figure 4B) is generated from band ratios 960 cm^−1^ (PO_4_^−3^)/1,460 cm^−1^ (CH_2_ wagging, amide II) and in this image, intact dentin is dark red while peptide-functionalized adhesive-infiltrated demineralized dentin is blue. Figure 4C is the K-means clustering map (DCA) of the Raman profile of the same scan area. With the assistance of the cluster analysis, a false color-image of the substrate, on the basis of similar spectral features, was produced. As the cluster centroids are essentially means of the cluster score for the elements of the cluster, ID and three regions on the left could be identified and examined for each cluster (Figure 4C).

In addition to the collagen features, the Raman spectral features associated with methacrylate-based adhesive appeared in these three regions, such as 605 cm^−1^ (C-COO),853 cm^−1^ (*v* CH_2_),1,084 cm^−1^ (*v* C-C), 1,450 cm^−1^ (CH def), 1,608 cm^−1^ (phenyl), 1,635 cm^−1^ (*v* *C* = C), and 1,710 cm^−1^ (*v* *C* = O). The band intensity of 1,635 cm^−1^ (*v* *C* = C) is quite weak indicating the infiltrated adhesive was polymerized well, i.e., high degree of monomer-to-polymer conversion. The spectral features associated with adhesive contributed less in the two small regions on the far left, thus these two small regions are identified as partially infiltrated demineralized dentin (P-PIDD). As a reference, Raman spectra of peptide-functionalized adhesive and co-polymerizable hydroxyapatite binding peptide (MMES-KGGG_HABP) are shown in Supplementary Figure 1. Raman spectra of intact dentin, demineralized dentin and peptide-functionalized adhesive-infiltrated demineralized dentin are shown in Supplementary Figure 2. Spectra acquired from calcium phosphate standards, i.e., hydroxyapatite, α-tricalcium phosphate, β-tricalcium phosphate, and amorphous calcium phosphate are shown in Supplementary Figure 4.

**FIGURE 5 |:**
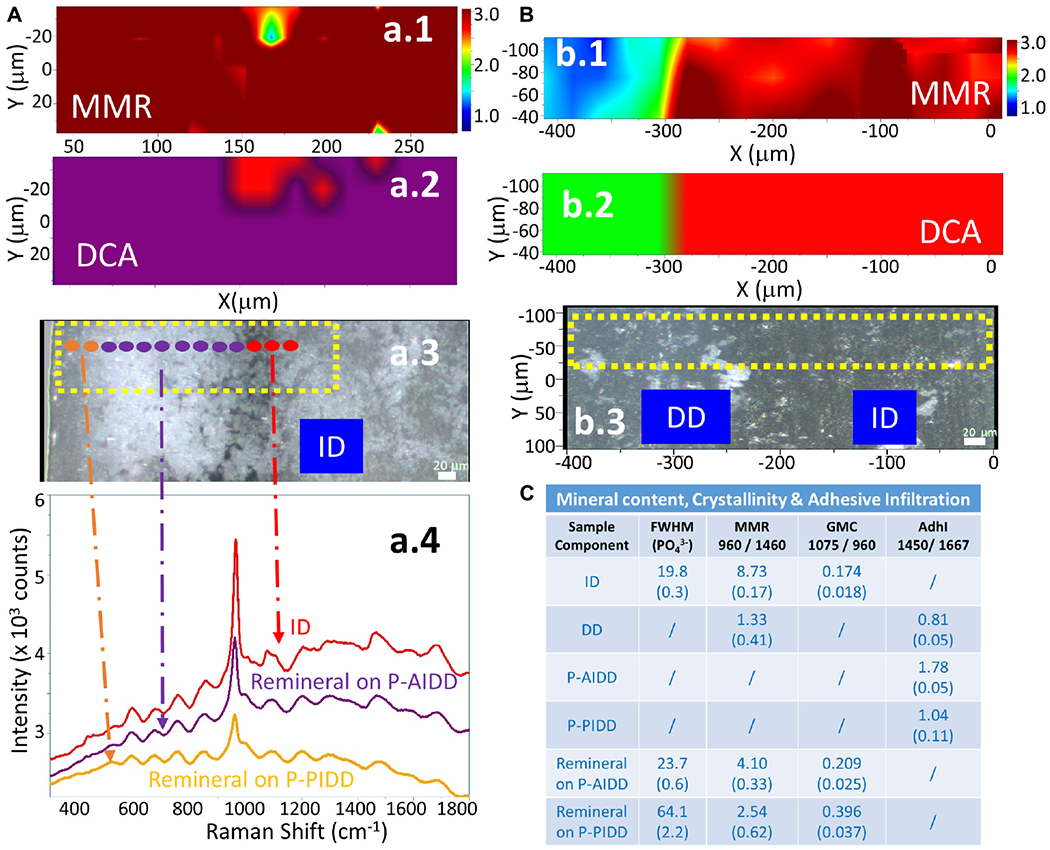
RAMAN analysis of a representative peptide-functionalized adhesive-infiltrated demineralized dentin (P-AIDD) specimen after remineralization **(A)** and a control demineralized dentin (DD) specimen after remineralization protocol **(B)**. (a.1,b.1) μ-Raman mineral map (MMR) of the X-Y scan area. Blue represents the lowest ratio, while red represents the highest. (a.2,b.2) K-means clustering map (DCA) of the Raman profile of the same sample. (a.3,b.3) Visible images of the scanned areas. Scale bar is 20 μm. (a.4) Raman spectra of principal components (PCs) from the DCA. **(C)** Summary of the Raman spectral features related to quantification of mineral content, crystallinity, and adhesive infiltration to the dentin samples before and after remineralization.

Raman images of the specimens following treatment with the remineralization protocol are presented in Figure 5. Analyses of representative peptide-functionalized-adhesive-infiltrated regions (P-AIDD) and control demineralized dentin specimens are presented in Figures 5A,B, respectively. The micro-Raman mineral map of a representative P-AIDD specimen (Figure 5Aa1) shows mineral throughout the area represented by peptide-functionalized-adhesive-infiltrated regions and extending into the intact dentin region. Following treatment with the remineralization protocol, the P-AIDD specimen demonstrated a layer of peptide-mediated mineral with distinct spectral features associated with *v*1 phosphate band at 960 cm^−1^ (Figure 5Aa4). In contrast, there is a distinct absence of the characteristic band associated with mineral (960 cm^−1^) in the control demineralized dentin specimen after the remineralization protocol (Figure 5b1). The cluster analysis verifies this result (Figure 5b2) showing the DD region (in green) and ID (in red). The visible images of the control demineralized dentin samples show very few hydroxyapatite crystals (white deposits) within the demineralized dentin region (Figure 5b3).

The cluster analysis provides further information about the peptide-mediated mineral in the P-AIDD specimen. Quantification of mineral content, crystallinity, and adhesive infiltration in the different dentin samples before and after treatment with the remineralization protocol, are summarized in Figure 5C. The calculated mineral content for ID (Figure 3D) and DD specimens is used as a reference. The MMR values were ~8.7 for ID, and 1.3 for DD (as base line), whereas the MMR values for the new peptide-mediated minerals are about 3~4. The relative crystallinities of the new peptide-mediated minerals are different from that of intact dentin, with their FWHM much larger than that of intact dentin (*p* < 0.05). Using FWHM as a measure of crystallinity, the different sample components can be ordered, e.g., ID > remineral on P-AIDD > remineral on P-PIDD specimen. The carbonate content of the new peptide-mediated remineralized dentin presented as GMC is also larger than that of intact dentin (*p* < 0.05), indicating the reverse order for the carbonate substitution for phosphate, e.g., ID < remineral on P-AIDD < remineral on P-PIDD specimen. The adhesive infiltration calculated from band ratio 1,450/1,667 verified that P-PIDD has less adhesive contribution (~1.0), compared to that of P-AIDD (~1.8).

### Atomic Force Microscopy

**FIGURE 6 |:**
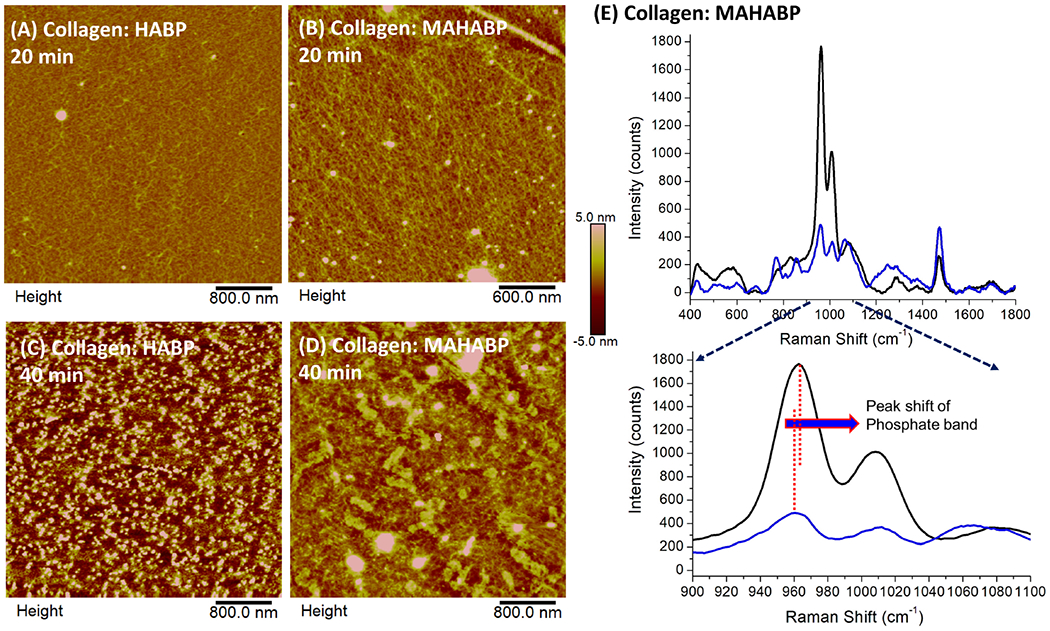
AFM images of peptide-functionalized collagen model surfaces after alkaline phosphatase mediated mineralization and their Raman spectra. **(A)** Collagen: HABP after 20 min; **(B)** collagen: MAHABP after 20 min **(C)** collagen: HABP after 40 min; **(D)** collagen:MAHABP after 40 min **(E)** Raman spectra of the minerals grown on the MAHABP-functionalized collagen model surfaces at 20 min and longer mineralization.

The collagen model specimens with presence of peptides were used to simulate the interface between demineralized dentin collagen and co-polymerizable HABP, i.e., MAHABP. Figure 6 shows early stages of peptide-mediated mineralization on the model specimens. The collagen fibrils are presented in Figures 6A,B with scattered mineral particles after 20 min peptide-mediated mineralization initiated. Mineral growth extended throughout collagen network after 40 min (Figures 6C,D). Subtle differences are spotted in the mineral growth between the HABP and co-polymerizable HABP, i.e., MAHABP. While collagen network could still be observed in the presence of HABP, MAHABP resulted in large mineral island formations. The Raman spectra of the mineral produced with collagen: MAHABP after 20 min was obtained and compared with that of later stage mineral (Figure 6E). Based on the spectral analysis, the early-stage mineral has less mineral concentration, larger FWHM (960 cm^−1^) and higher carbonate content. The *v*1 phosphate band shifted to a higher position at later stage of mineralization as seen in Figure 6E.

## DISCUSSION

### Conventional Raman Spectroscopy and Its Limitations

Overlapping features were observed in the spectra of the peptide-functionalized adhesive, intact dentin, demineralized dentin, and peptide-functionalized adhesive-infiltrated demineralized dentin (Supplementary Figures 1, 2). For instance, the CH_2_ wagging (1,460 cm^−1^) deformation bands in demineralized dentin collagen (generally assigned to proteins, lipids and carbon hydrates) overlap with the peak at 1,450 cm^−1^ (assigned to the C-H group of methacrylate-based adhesive). Due to the overlapping spectral bands, univariate analysis, such as mapping of peak areas of specific functional groups or band ratio values, do not always accurately identify the location and concentration of the functional group. Unanticipated components may be completely overlooked after data processing using univariate analysis.

In addition to the challenges associated with overlapping spectral bands, the non-uniform coverage of new peptide-mediated minerals on the surface of the experimental specimens may directly influence the point-to-point reproducibility of the recorded spectra. On a point-to-point basis, the spectral differences are often subtle, but they are key to differentiating composition and crystallinity. Faced with these challenges, the simplified peak area calculations with univariate analysis offered limited information—quantified relationships could not be determined using these calculations. The challenges associated with these specimens also made discriminating between species very difficult. Multivariate analysis tools were required to track subtle differences in these complex, heterogeneous samples.

### Insights Gained Through Combined Chemometric Analysis

Divisive clustering analysis (DCA) was selected for this study to separate the group of spectra into clusters with clear similarities, i.e., similarities within each cluster and distinctions between the clusters. The biochemical content of each cluster, e.g., the mineral and organic components of the adhesive/collagen hybrid, was analyzed using the average cluster spectra. These multivariate analyses confirmed the differences in the spectral composition. Separation of chemical spectra distinct to each chemical constituent is inherent in the algorithm, thus digital subtraction of spectra with overlapping features is not needed—digital subtraction can obscure subtle differences. The cluster analyses revealed detailed differences in the P-AIDD specimen (Figure 4C), where, four different components, i.e., ID, P-AIDD and two P-PIDD regions correspond to the four clusters that were created. All spectra for each cluster were averaged to obtain the mean cluster spectrum, for each point of analysis (Figure 4D).

Different levels of the C-H bond (at 1,450 cm^−1^) associated with adhesive were noted at the DCA centroids associated with P-AIDD and the two P-PIDD regions. These differences were not visible following univariate analyses (Figure 4B). Indeed, after cluster analysis of the area as a whole and over the full spectral range, each principal component represents one or two chemical constituents in the specimen that are uncorrelated with other constituents (Figure 4C). This means that the spectra are classified using a systematic approach, which directly offers information about the distribution of components in the specimen, as opposed to univariate methods that involve a more trial-and-error procedure along with the necessity for *a priori* knowledge of the specimen.

### Mineral Content

The additional insights gained through this chemometrics analysis include the relative mineral content, carbonate content and the mineral crystallinities. This information was revealed in the average cluster spectra. Crystal formation was not encountered in the control group after the remineralization protocol, and their mineral-to-matrix ratio (MMR) values did not change, being 1.3 as base line (*p* > 0.05). The existence of new peptide-mediated mineral following treatment with the remineralization protocol is a crucial finding with the peptide-functionalized adhesive-infiltrated demineralized dentin (P-AIDD) samples. The mineral crystals suggest that the peptide-functionalized adhesive is responsive to environmental stimuli, in this case mineralization buffer.

### Crystallinity

Vibrational spectroscopic techniques such as FTIR and Raman, are used intensively to determine crystal phases of mineralized materials (Toledano et al., 2012). The band width, i.e., full-width-half-maximum (FWHM) and Raman shift of *v*1 phosphate band are used to indicate the structural differences in mineral. In general, the narrower the spectra peak width, the higher the degree of mineral crystallinity (Karan et al., 2009).

The PO_4_ band in spectra of the new peptide-mediated mineral in the P-AIDD region of the sample is narrower and more resolved than the PO_4_ band in the P-PIDD region (Figures 5a3,C). This difference suggests less structural variation in bond distances and angles in the peptide-mediated mineral in the P-AIDD region (Schwartz et al., 2012). This effect shows a higher degree of crystallinity of the new mineral in the P-AIDD region as compared to the new peptide-mediated mineral in the P-PIDD region. This was probably due to less incorporation of peptide-functionalized adhesive in the P-PIDD region compared to that in the P-AIDD region. These differences support the potential of tailoring the peptide-functionalized adhesive to the tissue interface.

Raman shift of *v*1 phosphate band center usually identifies the classification of a crystal feature. This *v*1 phosphate band of normal dentin and bone is usually located at 950–960 cm^−1^ and this band could be shifted to a position close to 970 cm^−1^ for β-tricalcium phosphate (β-TCP) (Penel et al., 2005). In the fractured specimen, the *v*1 phosphate band of intact dentin was located at 964 cm^−1^. Investigators reported that in the micro-damaged and fractured regions of mineralized tissue, the *v*1 phosphate band shifted to a higher wavenumber (963 and 965 cm^−1^, respectively) than the *v*1 phosphate band in carbonated hydroxyapatite associated with tooth or bone (959 cm^−1^) (Dooley et al., 2009). The Raman shift of *v*1 phosphate band center for the new peptide-mediated minerals in this study is very close to that for chemically synthesized hydroxyapatite (960 cm^−1^) (Daculsi et al., 1987; Duke and Lindemuth, 1991).

### Carbonate Content

Biological apatite is often calcium deficient with substantial amounts of carbonate occupying the phosphate position in the hydroxyapatite structure (Zurick et al., 2013). Carbonated apatite is a precursor of hydroxyapatite. The *v*1 carbonate band at ~1,070 cm^−1^ in the Raman spectra is often used to measure the relative B-type carbonate content (Awonusi et al., 2007). Detailed analysis of the average cluster spectra data suggested that the new peptide-mediated mineral has relatively higher carbonate content (Figure 5C), and the carbonate content on the P-AIDD and P-PIDD regions are distinctly different. The FWHM of *v*1 phosphate and the carbonate content are correlated, e.g., the higher the carbonate content in the mineral, the broader the *v*1 phosphate band. This means lower mineral crystallinity yields wider phosphate band, which is mirrored by high carbonate substitution (i.e., increased carbonate-to-phosphate ratio) in the present study. The structural differences in the peptide-mediated mineral associated with the P-AIDD and P-PIDD provide evidence that the peptide-functionalized adhesive is responsive to environmental stimuli. These structural differences also support the potential of tailoring the peptide-functionalized adhesive to the tissue interface. The results support the potential of engineering the adhesive to promote peptide-mediated remineralization at the adhesive/dentin interface.

### Defective Material/Tissue Interface and Our Bio-Enabled Approach to Address This Weakest Link

In our previous investigations, we used micro-scale structure/property measurements as a guide to develop an idealized microstructural representation of the hybrid layer (Wang and Spencer, 2003; Singh et al., 2015). The adhesive/collagen hybrid constructs were prepared using a hydrophobic dental adhesive and collagen matrix from demineralized bovine dentin. Time-dependent and rate-dependent mechanical behavior of this ideal adhesive/collagen hybrid was investigated under conditions that simulated the functional environment of the mouth. The results emphasized the complexity of the interfacial behavior. For example, the ideal adhesive/collagen hybrid experienced smaller and uniform stress due to constant material properties along the depth of the hybrid layer (Spencer and Misra, 2017), but the behavior depended on several factors including moisture content, adhesive characteristics, relative ratio of adhesive and collagen, loading level and loading rate. Fatigue was affected by both the material components and micro-structure (Singh et al., 2015).

The complex structure/property relationships that determine durability at the adhesive/dentin interface are magnified under clinical conditions. The adhesive seal to dentin is fragile and the fragility can be traced to defects such as pockets of resin-sparse collagen—this unprotected collagen is degraded by acids and enzymes. Bacteria and bacterial by-products infiltrate the resulting interfacial gaps, demineralize and decompose the tooth, and further erode the adhesive, leading to wider and deeper gaps that create an ideal environment for bacteria to proliferate. These activities lead ultimately to recurrent decay, hypersensitivity and pulpal inflammation (Van Meerbeek et al., 2005; Nedeljkovic et al., 2015, 2016; Spencer et al., 2019). The current investigation addresses this problem by using a peptide-functionalized adhesive to promote peptide-mediated remineralization of interfacial defects—defects that maybe traced to resin-sparse pockets of collagen.

The remineralization experiment was initiated using enzyme-based assay mimicking biological systems by incorporating alkaline phosphatase enzyme. This enzyme-based mineralization offers control of the kinetics by controlling the phosphate ion release from the organic phosphate source cleaved by alkaline phosphatase. Dentin remineralization is driven by mineral growth within nucleation sites in preserved collagen fibrils (Bertassoni et al., 2011), however, the role of the collagen matrix in dentin remineralization is still controversial. It was reported that crystal apatites remaining on demineralized collagen fibrils can serve as nucleation templates or seeds (Zhang et al., 2003).

We propose that the remineralization observed with P-AIDD is a result of 2-phase sequence, i.e., binding to the exposed collagen fibers and crystal nucleation facilitated by the tethered mineral-forming peptide. The tethered mineral-forming peptide may offer preferential binding sites for ions (calcium, phosphate, fluoride, and hydroxyl ions), chelate ions and promotes mineralization through controlling kinetics. Overall the peptide conformation and available sites for chelated ions and their binding kinetics in the collagen network could serve as a template for peptide-assisted mineral formation.

The control demineralized dentin specimens treated with the remineralization protocol showed few, tiny, scattered mineral crystals, and the spectral features associated with mineral-derived bands were very weak. The diminished remineralizing effect might be linked to poor mineral precipitation of calcium and phosphate ions at the demineralized organic matrix (McKee et al., 2011).

### Initial Mineralization at the Collagen Interface

Based on the early-stage mineralization study, mineral particles (50–300 nm) were detected by AFM after only 20 min reaction (Figure 6). The results suggest that mineral growth was quicker and more uniform with the co-polymerizable hydroxyapatite-binding peptide (MAHABP) on the collagen model specimens (Figure 6B). With longer mineralization time, e.g., 40 min, the mineral particles grow rapidly and start to form clusters (Figures 6C,D).

The broad and poorly resolved PO_4_ spectral features suggest reduced atomic order, i.e., more structural variation in bond distances and angles, in the early-stage mineral (Figure 6E). The PO_4_ band was narrower and better resolved after the longer mineralization reaction; these spectral results indicate a higher degree of crystallinity. The carbonate content also decreased after the longer mineralization reaction. The relationship between the crystallinity and carbonate content in this mineral maturation process is similar to the case of P-AIDD study.

### Limitations and Next Steps

The dentin collagen matrix treated with the peptide-functionalized adhesive showed promising results in terms of delivering biologic cues direct at the adhesive/dentin interface. There are, however, limitations that require further investigation. Raman is a surface-sensitive technique and thus, mineral formation was demonstrated at the surface. Further research is needed to determine the depth of the remineralization reaction. In addition, the kinetics of nucleation, crystal formation, and time-resolved compositional changes must be determined. Based on initial light microscopic analysis, mineral crystals started to grow on the surface of P-AIDD specimens at about 24–30 h (unpublished results). In addition, the amount of co-polymerizable peptide used in the adhesive formulation could be a major factor for the remineralization reaction. In the current investigation, 10 mass% of co-polymerizable HABP was used. Future investigations should include detailed studies on the peptide mediated mineralization kinetics and determine the parameters affecting the kinetics under clinically relevant conditions.

The factors that led to the P-PIDD regions as the top layer of the peptide-functionalized-adhesive-infiltration demineralized dentin have not been resolved in this investigation. These studies should be extended to optimize the protocol. For example, these P-PIDD regions maybe related to sample preparation, e.g., the air stream which was forced across the surface of the specimen may have dislodged the relatively hydrophilic, low viscosity adhesive. In addition, the surface layer of this hydrophilic resin may not have been adequately polymerized due to oxygen inhibition (Ruggeberg and Margeson, 1990). Under these circumstances, the unpolymerized monomer could be leached which would reduce infiltrated adhesive. The depth of P-PIDD region is about 50 μm which is close to the depth of oxygen inhibition layer reported in the literature (Ruggeberg and Margeson, 1990; Nunes et al., 2005).

### Understanding Reactions at Interface and Knowledge-Based Engineering Biomaterials

Our prior work used a peptide-based approach to build an engineered fluorescent probe to label mineralized tissues (Yuca et al., 2011), developed a biomarker protein tag on the mineral forming peptide (Ye et al., 2017), and designed a biomimetic adhesive formulation using a combination of peptide and a ε-poly-lysine resin system (Xie et al., 2019). To address concerns associated with non-specific interaction, a peptide system capable to mediate mineralization, to incorporate a spacer sequence to retain active peptide conformation, and conjugated with a methacrylate-based monomer for copolymerization into dental adhesive polymer was designed and synthesized in the current work. This engineered peptide-based copolymer is a promising approach for a next generation adhesive to repair deficient dentin matrices and protect exposed collagen at the vulnerable adhesive/dentin interface.

## CONCLUSION

For the first time, we introduce a combined Raman spectroscopy and chemometrics approach to investigate heterogeneous material/tissue interfaces where biological cues were provided to promote tissue mineralization of deficient dentin matrices. The approach correlates physical properties to analytical data by extracting important information hidden in the heterogeneous interfaces. The adhesive/collagen hybrid served as a model of the heterogeneous adhesive/dentin interface that is formed during composite restoration. We extended our analyses to next generation adhesive design where remineralization of exposed collagen at the vulnerable adhesive/dentin interface was achieved using a stimuli-responsive engineered peptide-functionalized adhesive.

Analysis of these hybrid systems using Raman spectroscopy in combination with chemometrics revealed important structural details about the mineral content, crystallinity and carbonate content. The existence of new peptide-mediated mineral crystals is a crucial finding with the peptide-functionalized adhesive-infiltrated demineralized dentin (P-AIDD) samples. The peptide-mediated mineral crystals suggest that the peptide-functionalized adhesive is responsive to environmental stimuli, in this case mineralization buffer. There is a higher degree of crystallinity in the new mineral formed at the region of the specimen marked by complete infiltration with the peptide-functionalized adhesive as compared to partial infiltrate. These differences are also reflected in the carbonate content, i.e., the lower mineral crystallinity noted with partial infiltration is associated with increased carbonate-to-phosphate ratio. This was probably due to less incorporation of peptide-functionalized adhesive in the partially infiltrated demineralized dentin (P-PIDD) region compared to that in the adhesive-infiltrated demineralized dentin (P-AIDD) region. These differences support the response of the peptide-functionalized adhesive to environmental stimuli and the potential of tailoring the peptide-functionalized adhesive to the tissue interface.

Chemometrics-assisted confocal Raman spectroscopy provides more accurate information on the chemical composition and enhanced analyses of the mineral structure. Micro-Raman spectroscopy coupled with multivariate data analysis facilitated straightforward and efficient *in situ* structural characterization of these complex, heterogeneous material/tissue interfaces.

Overall, our results demonstrate that essential information hidden in the complex material/tissue interfaces, both structural and compositional information can be extracted using Raman spectroscopy combined with chemometrics. The insight provided by this data-driven approach can improve our understanding of phenomena that lead to failure at the biomaterial/tissue interface. This knowledge will enhance our ability to identify features within the interfacial micro-environment that should be harnessed to engineer biomaterials that will integrate with biological systems to promote tissue health and healing.

## Supplementary Material

Supplementary Material

## Data Availability

The original contributions presented in the study are included in the article/Supplementary Material, further inquiries can be directed to the corresponding author/s.

## Abbreviations:

AdhI: Adhesive Infiltration
DCA: divisive clustering analysis
DD: Demineralized Dentin
FWHM: Full-width half-maximum
GMC: Gradient in Mineral Content
HABP: hydroxyapatite-binding peptide
ID: Intact Dentin
MMES: mono(2-methacryloyloxy)ethyl succinate
MMR: Mineral-matrix Ratio
P-AIDD: (Peptide-functionalized-adhesive)-Adhesive-Infiltrated Demineralized Dentin
P-PIDD: (Peptide-functionalized adhesive)-Partially-Infiltrated Demineralized Dentin
