# Supplementary material for: Chemometrics-Assisted Raman Spectroscopy Characterization of Tunable Polymer-Peptide Hybrids for Dental Tissue Repair
Source: Front Mater. Author manuscript; Available in PMC 2021 Nov 1. (PMC8186416; doi:10.3389/fmats.2021.681415)
Supplement: Supplementary Material [file NIHMS1704695-supplement-Supplementary_Material.pdf]

Supporting Information

Raman spectra

The lyophilized peptide powders of copolymerizable hydroxyapatite binding peptide (MMES-KGGG_HABP) and the polymerized peptide-functionalized adhesive samples were imaged using a LabRAM ARAMIS Raman microscope (HORIBA Jobin Yvon, Edison, NJ). Raman spectrum of MMES-KGGG_HABP contained the spectral bands of 1337, 1634, and 1669 cm^{-1} characteristic of an α-helix conformation (Fig. S1). It also contained the spectral bands of 1428, and 1264 cm^{-1} characteristic of the CH_2CH_3 deformation and a β-strand conformation, respectively. The α-helix secondary structure is a design target for functional integration of the peptide to the adhesive system.

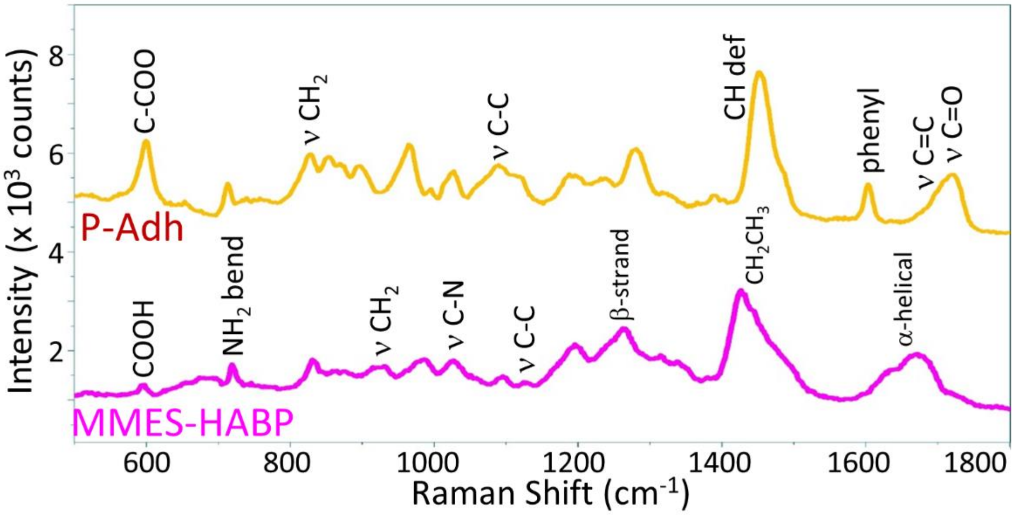

Figure S1. RAMAN spectra of the peptide-functionalized adhesive (P-Adh), and the co-polymerizable hydroxyapatite binding peptide (MMES-HABP).

In the Raman spectrum of the peptide-functionalized adhesive (P-Adh), the spectral features were basically associated with the methacrylate, such as 605 cm^{-1} (C-COO), 853 cm^{-1} (ν CH_2), 1084 cm^{-1} (ν C-C), 1455 cm^{-1} (CH def), 1608 cm^{-1} (phenyl), 1635 cm^{-1} (ν C=C) and 1710 cm^{-1} (ν C=O) (Fig. S1). Since the amount of the peptide monomer used in the adhesive formulation was less than 10%, the contribution of spectral features of peptide was not obvious in this Raman spectrum.

Chemometrics

Chemometrics is used as a quantitative chemical assessment methodology since the Raman peak intensity is proportional to the number of the molecules within the volume of the scanned area. (Adams, 1995; Jetter et al., 2000; Blat et al., 2019; Kopec et al., 2019; Abramczyk et al., 2020) The success of chemometrics approach, used in this study, stems from its ability to extract only the essential and relevant portions of the data for analysis, after filtering out noise and unnecessary information. This ability is essential when component separation for analysis is not possible, e.g., in situ analyses of material/tissue interfaces. Overlapping features were apparent in the spectra of the peptide-functionalized adhesive (Fig. S1), intact dentin, demineralized dentin, and peptide-functionalized adhesive-infiltrated demineralized dentin (Fig. S2).

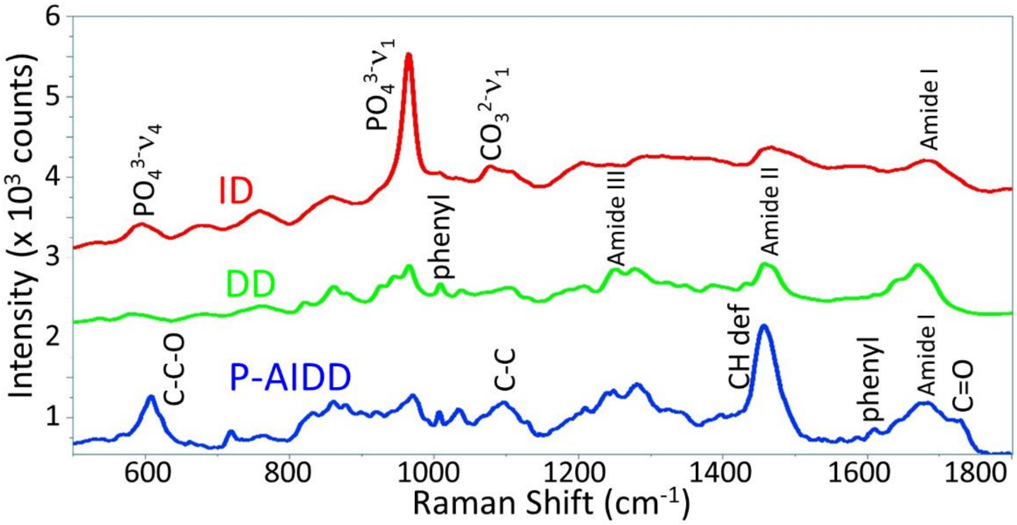

Figure S2. RAMAN spectra of intact dentin (ID), Demineralized Dentin (DD) and peptide-functionalized adhesive-infiltrated demineralized dentin (P-AIDD).

Divisive Clustering Analysis (DCA) uses K-means (the spectra are partitioned into k clusters so that each object belongs to the cluster with the nearest mean) and involves iterative cycles to find the most relevant groupings. This is an unsupervised method, but choice of the number of classes is critical. Fig. S3 gives an example, where the number of clusters, four, were selected for the adhesive-infiltrated demineralized dentin specimen, based on the false-color maps and the cluster centroid. Fig. S3A shows the data separated into four clusters. A false color image was obtained on the basis of similar spectral features after assigning each cluster to a different color (Fig. S3B). Four different components, e.g., ID, P-AIDD, and two P-PIDD regions corresponded to the four clusters. All spectra described for each cluster were averaged to obtain the mean cluster spectrum, for each point of analysis (Fig. S3C).

Four distinct centroids that reflected difference in Raman spectral distribution and chemical composition (in terms of mineral, collagen and adhesive components) corresponded to the DCA Raman images. For instance, different levels of the C-H bond (at 1450 cm^{-1}) associated with adhesive were evident at the DCA_1, DCA_2 and DCA_3 centroids (Fig. S3C). A separate region where there is more mineral contribution, DCA_4 centroid (appears red in Fig. S3B) represents 35% of the variance. Further clustering (more than four) results in the formation of duplicate clusters indicating complete overlap and similarity between both measurements, which means inclusion of more principal components does not introduce significant changes in the clusters. As a result, the number of clusters as well as factors was fixed at four.

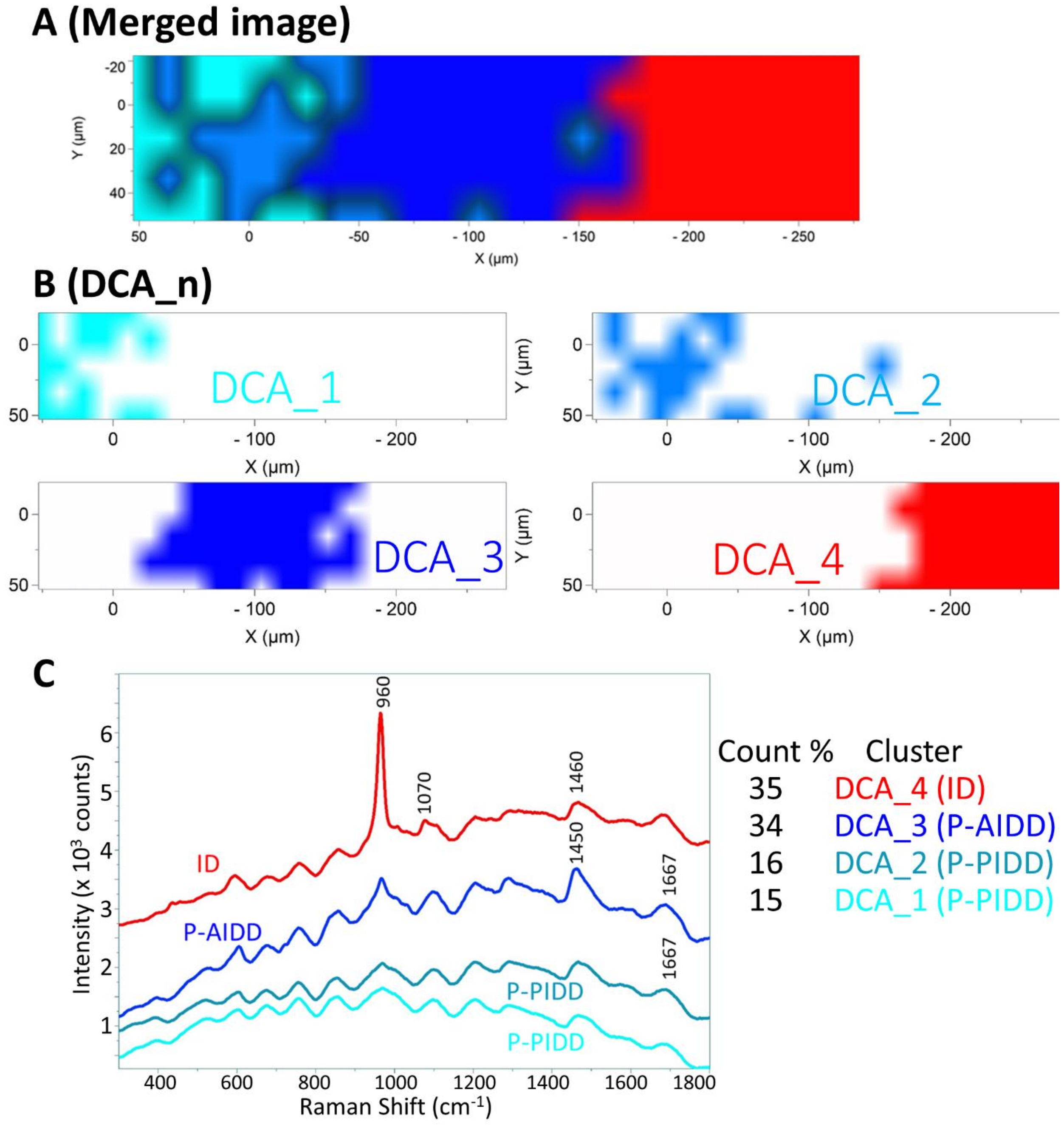

Figure S3. Color mapping from divisive clustering analysis (DCA) corresponding to the peptide-functionalized adhesive-infiltrated demineralized dentin (P-AIDD). Four levels DCA clustering images are shown and grouped into 4 clusters, each cluster corresponding to a set of Raman shift. Areas of distinct color have differences in Raman spectral distribution and chemical composition. Each centroid cluster is assigned to a different color, thus obtaining a false color-image on the basis of similar spectral features. (A) a merged image from the four centroid clusters. Upon attributing different colors to each centroid image (B) and setting the color scale so that intensity colored region (i.e. pixels having high intensity value) are enhanced over "white" region (i.e. pixels having medium-low relative intensity value), these can be merged into

a single image depicting the spatial distribution of different chemical species, each color corresponding to the set of Raman shifts characterizing that chemical species. (C) average Raman spectra of the four classes, showing the assignments for dentin mineral and collagen matrix-specific Raman bands, in the region from 300 to 1800 cm^{-1}.

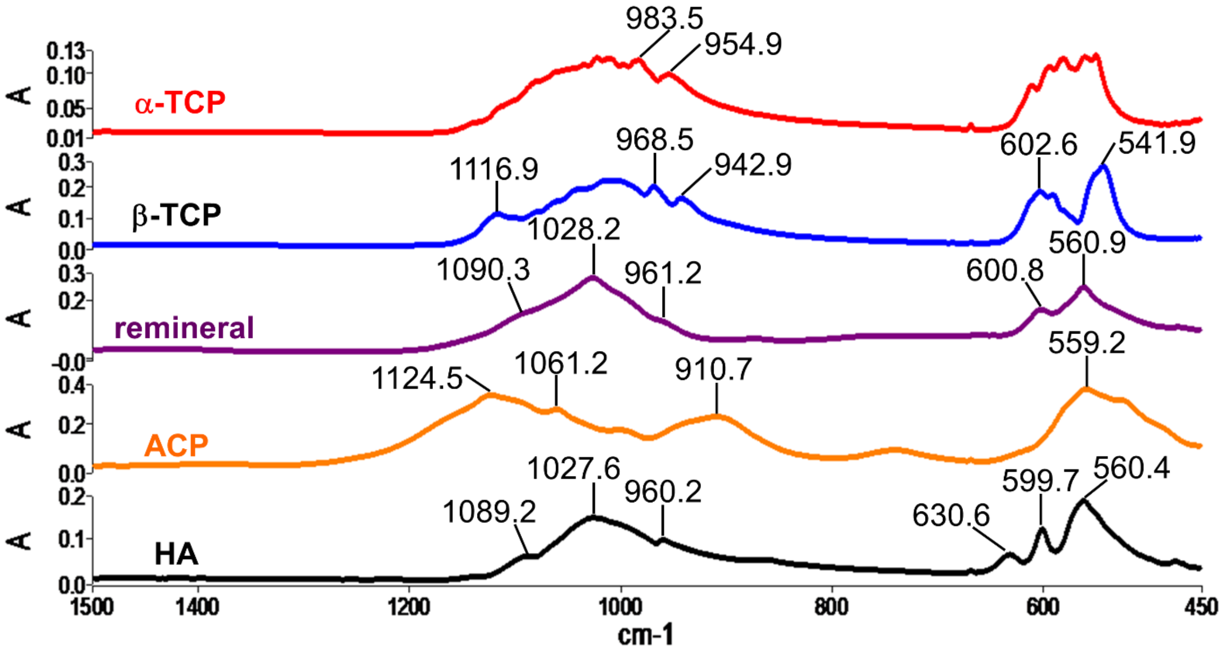

Figure S4. ATR/FTIR spectra of commercially available mineral standards, e.g., hydroxyapatite (HA), α-tricalcium phosphate (α-TCP), β-tricalcium phosphate (β-TCP), and amorphous calcium phosphate (ACP). Spectra were acquired at 4000-400 cm^{-1} , but the range 1500-450 cm^{-1} was selected to highlight the two intense adsorption peaks of $\nu_3(PO_4^{3-})$ and $\nu_4(PO_4^{3-})$.

ATR/FTIR spectra

FTIR spectroscopy was used to characterize the calcium phosphate phase in the mineral samples. These studies were performed using an infrared spectrometer (Spectrum 400, Perkin-Elmer, Waltham, MA) at a resolution of 4 cm^{-1} with 256 scans. Sample was placed on the crystal top plate of an attenuated total reflectance (ATR) accessory (PIKE Technologies Gladi-ATR, Madison, WI) and pressure was applied to maintain good contact between sample and the crystal. ATR/FTIR spectra of some calcium phosphate standards (commercially available hydroxyapatite, α-tricalcium phosphate, β-tricalcium phosphate, and amorphous calcium phosphate) and the peptide-assisted mineral formed on the P-AIDD samples were acquired (Figure S4). Characteristic splitting between phosphate ν_3 band (1028, 1090 cm^{-1}) and triply degenerated ν_4 (P-O-P bond, out-of-plane bending) at 560/575/600 cm^{-1} are shown in the spectra of the peptide-assisted mineral formed on the P-AIDD samples. These spectral features are typical for nanocrystalline apatites. Broad band around 560 cm^{-1} and absence of peak splitting around 1050 cm^{-1} are shown in the ACP spectra, typical for non-apatitic disordered state. Based on comparisons among these spectra, the formed mineral is identified as apatite crystals.

REFERENCES

Abramczyk, H., Brozek-Pluska, B., Jarota, A., Surmacki, J., Imiela, A., and Kopec, M. (2020). A look into the use of Raman spectroscopy for brain and breast cancer diagnostics: linear and non-linear optics in cancer research as a gateway to tumor cell identity. *Expert Review of Molecular Diagnostics* 20(1)**,** 99-115. doi: 10.1080/14737159.2020.1724092.

Adams, M.J. (1995). *Chemometrics in Analytical Spectroscopy.* Cambridge, UK: The Royal Society of Chemistry.

Blat, A., Dybas, J., Chrabaszcz, K., Bulat, K., Jasztal, A., Kaczmarska, M., et al. (2019). FTIR, Raman and AFM characterization of the clinically valid biochemical parameters of the thrombi in acute ischemic stroke. *Sci Rep* 9(1)**,** 15475. doi: 10.1038/s41598-019-51932-0.

Jetter, K., Depczynski, U., Molt, K., and Niemoller, A. (2000). Principles and applications of wavelet transformation of chemometrics *Anal. Chim. Acta* 420**,** 169-180.

Kopec, M., Imiela, A., and Abramczyk, H. (2019). Monitoring glycosylation metabolism in brain and breast cancer by Raman imaging. *Scientific Reports* 9. doi: ARTN 166 10.1038/s41598-018-36622-7.
